# Protocol for a systematic review on inequalities in postnatal care services utilization in low- and middle-income countries

**DOI:** 10.1186/2046-4053-2-55

**Published:** 2013-07-06

**Authors:** Étienne V Langlois, Malgorzata Miszkurka, Daniela Ziegler, Igor Karp, Maria Victoria Zunzunegui

**Affiliations:** 1 Research Centre of the University of Montreal Hospital Centre (CRCHUM), 3875 St-Urbain St, Montreal, QC H2W 1V1, Canada; 2 Department of Social and Preventive Medicine, University of Montreal, Pavilion 7101, Parc Avenue, Montreal, QC H3N 1X7, Canada; 3 Documentation Center of the University of Montreal Hospital Centre (CHUM), Saint-Luc Hospital, 1058, Saint-Denis St., Principal Pavilion, 1st floor #1303, Montreal, QC H2X 3J4, Canada; 4 University of Montreal’s Public Health Research Institute (IRSPUM), Pavilion 7101, Parc Avenue, C.P. 6128, Succ. Centre-Ville, Montreal, QC H3C 3J7, Canada

**Keywords:** Postnatal care, Health services utilization, Maternal health, Low- and middle-income countries, Inequalities, Systematic review, Protocol

## Abstract

**Background:**

Each year, 287,000 women die from complications related to pregnancy or childbirth, and 3.8 million newborns die before reaching 28 days of life. The near totality (99%) of maternal and neonatal deaths occurs in low- and middle-income countries (LMICs). Utilization of essential obstetric care services including postnatal care (PNC) largely contributes to the reduction of maternal and neonatal mortality and morbidity. There is a strong need to evaluate the evidence on the unmet needs in utilization of PNC services to inform health policy planning. Our objective is to assess systematically the socioeconomic, geographic and demographic inequalities in the use of PNC interventions in low- and middle-income countries.

**Methods/Design:**

The current protocol adopts a strategy informed by the guidelines of *The Cochrane Handbook for Systematic Reviews*. Our systematic review will identify studies in English, French, Spanish, Portuguese and Chinese – provided inclusion of an English abstract - from 1960 onwards, by searching MEDLINE (PubMed interface), EMBASE (OVID interface), Cochrane Central (OVID interface) and the gray literature. Study selection criteria include research setting, study design, reported outcomes and determinants of interest. Our primary outcome is the utilization of PNC services, and determinants of concern are: 1) socioeconomic status (for example, income, education); 2) geographic determinants (for example, distance to a health center, rural versus urban residence); and 3) demographic determinants (for example, ethnicity, immigration status). Screening, data abstraction, and scientific quality assessment will be conducted independently by two reviewers using standardized forms. Where feasible, study results will be combined through meta-analyses to obtain a pooled measure of association between utilization of PNC services and key determinants. Results will be stratified by countries’ income levels (World Bank classification).

**Discussion:**

Our review will inform policy-making with the aim of decreasing inequalities in utilization of PNC services. This research will provide evidence on unmet needs for PNC services in LMICs, knowledge gaps and recommendations to health policy planners. Our research will help promote universal coverage of quality PNC services as an integral part of the continuum of maternal and child health care. This protocol was registered with the Prospero database (registration number: CRD42013004661).

## Background

Each year, 287,000 women die while pregnant, during childbirth or within 42 days of termination of pregnancy [1]. Maternal mortality mostly occurs within childbirth and the first week postpartum [2], and more than half (56%) of the world’s maternal deaths are recorded annually in Sub-Saharan Africa [1]. The annual toll of losses resulting from poor pregnancy outcomes further includes more than three million stillbirths - of whom at least one million die during labor - and 3.8 million neonatal deaths (decease of the live newborn within 28 days) [3]. Ninety-nine percent of maternal deaths and the same percentage of neonatal deaths occur in low- and middle-income countries (LMICs), where a large proportion of births take place at home and where postnatal care (PNC) for mothers and neonates is either not available or is of poor quality [1,4]. Sub-Saharan Africa accounts for 38% of global neonatal deaths and records the highest neonatal mortality rate in the world (34 deaths per 1,000 live births in 2011) [5].

It is largely acknowledged that utilization of essential obstetric care services - including but not limited to antenatal care, skilled attendance at birth and postnatal care - contribute to the reduction of maternal and neonatal mortality and morbidity in LMICs [6,7]*.* The fifth United Nations Millennium Development Goal aims to achieve universal access to reproductive health services by 2015, including coverage of obstetrical care services [8]. In this context, policy makers, development agencies and researchers are showing increased interest in access to and utilization of PNC services provided by skilled health professionals.

The World Health Organization (WHO, 2010) stated that the postnatal period begins immediately after the birth of the baby and extends up to six weeks (42 days) after birth [9]. The principal objectives of PNC services are to evaluate, maintain and promote the health of the birthing woman and the newborn and to foster an environment that offers help and support for diverse health and social needs. Follow-up visits include the evaluation of the parturient health status including screening, diagnosis and treatment of various conditions: tuberculosis, malaria, vaginal infections, anemia or malnutrition [10]. Assuring high nutritional intake - iron and calcium fortified diets - during the postpartum period counteracts anemia and provides calories for adequate milk production [11]. In the context of LMICs, PNC services include health education concerning early and exclusive breastfeeding (EBF) for a period of six months, as well as promotion of lifesaving interventions such as Kangaroo Mother Care for low birth weight (LBW) and premature babies, or using insecticide-treated bed nets to prevent malaria [12]. PNC services include counseling on available contraception, birth spacing and family planning options, along with diagnosing postpartum depression, often much neglected in LMICs.

PNC of the newborn covers screening and treating infections (signs include fever, respiratory distress, lethargy), jaundice and postnatal growth restriction, as well as dispensing immunization services and umbilical cord care [13]. Preterm, LBW, and HIV-infected newborns need special care in the postnatal period. Furthermore, PNC visits provide education of the mothers and families on seeking care for the baby upon noticing dangers signs, such as persistent vomiting, convulsions or not suckling. PNC services offer assessment of postnatal factors predisposing to anemia in infants and young children [14], and promote utilization of child health cards, inherently favoring infant health and compliance with the immunization schedule [15].

The number and timeliness of postnatal consultations are being studied at present by WHO to update clinical guidelines [9]. Some suggest a minimum of three PNC visits, one in the immediate postnatal period (first 24 hours from birth), another in the early postnatal period (days 2 through 7) and a third PNC in the late postnatal period (days 8 through 42) [9]. Different practice of PNC is observed; Uganda, for instance, is promoting follow-up of the mother and her baby by a skilled heath worker at six hours, six days and six weeks [16].

There are important unmet needs in PNC in LMICs, where more than 70% of all babies born outside the hospital do not receive any PNC services [17]. In a study conducted in 30 LMICs involving home and facility deliveries, an average of 40% of all women with a live birth in the previous five years did not receive any postpartum care check-ups [9,18]. In the absence of postnatal follow-up, numerous cases of puerperal infections go undiagnosed and unreported [19,20]. Most postpartum infections take place after hospital discharge, which is usually 24 hours after an institutional delivery. Furthermore, rates of provision of skilled care are lower during the postnatal period than during pregnancy or childbirth. Among women who did receive PNC, health professionals reportedly provided 57% of PNC services. The remainder received PNC from traditional birth attendants (36%) and others (7%) [18].

Scientific evidence exists on inequalities in the use of antenatal care (ANC), location of childbirth (home or facility delivery) and skilled birth attendant (SBA) at delivery across socioeconomic status [6,21], education [6,22], distance to a health center [6,23], and households located in urban versus rural areas [7,24]. Health seeking behavior for ANC and SBA services are stronger among educated, urban and higher socioeconomic status (SES) women, along with households living within 5 km of a health center [6,7,25,26]. However, knowledge is limited on the determinants of PNC services utilization. There is a strong need to evaluate systematically the existing evidence on inequalities in PNC services utilization on which to base health policy planning.

### Objectives and research questions

Our objectives are to: 1) systematically identify and assess studies and reports on the utilization of PNC services in LMICs; 2) synthetize evidence on the determinants of PNC services and inequalities in the use of PNC interventions in LMICs; and 3) provide evidence to policy planners in order to address unmet needs for PNC services in LMICs. This systematic review is guided by the following research questions: is PNC service utilization associated with 1) socioeconomic, 2) geographic and 3) demographic determinants?

## Methods/Design

**Figure 1:**
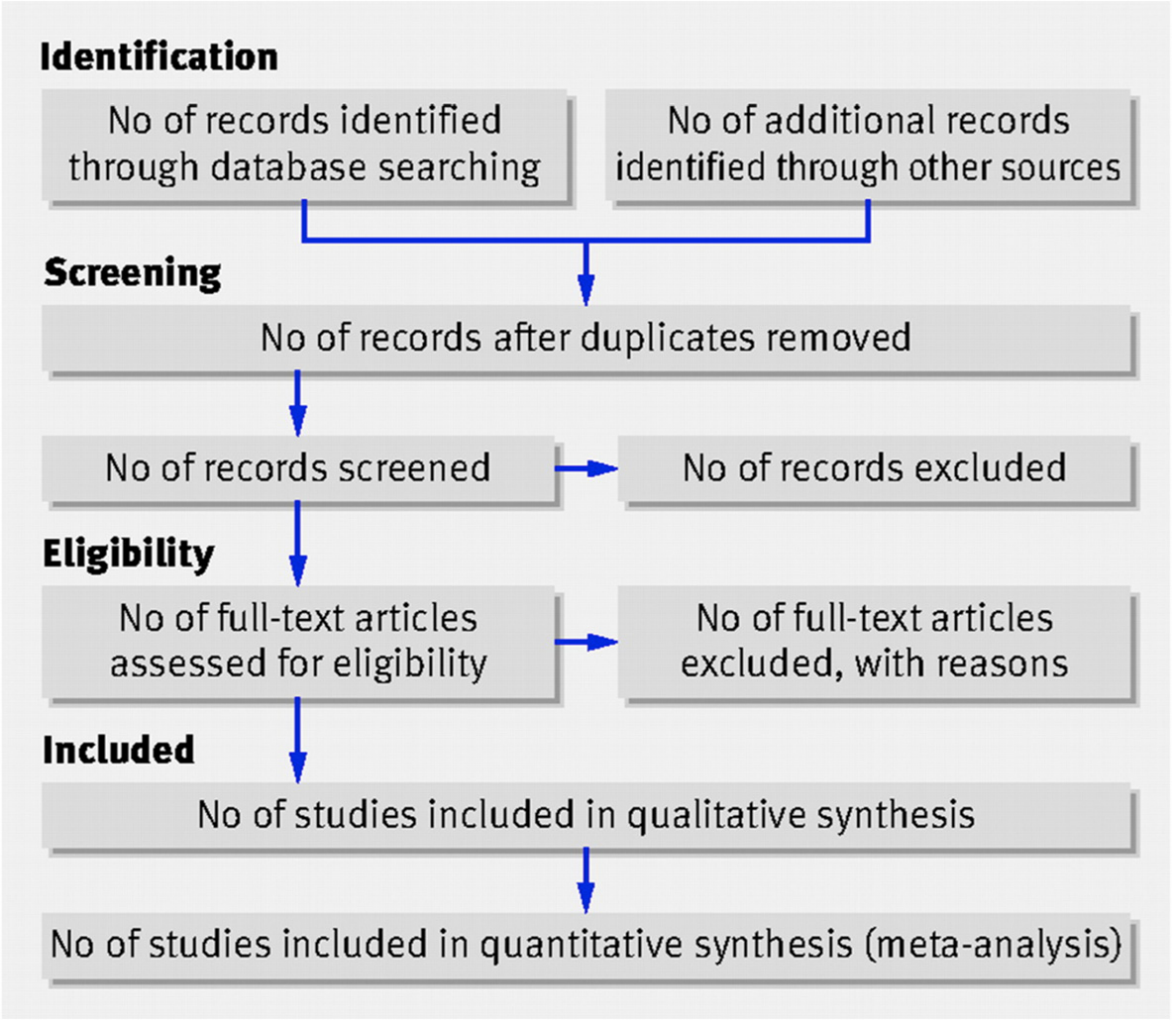
**PRISMA Flow Diagram Moher, D., *et al*. **[28]**.**

The current protocol outlines a strategy informed by the guidelines of The Cochrane Collaboration (*Cochrane Handbook for Systematic Reviews*) [27]. The systematic review will follow the four-phase flow diagram (Figure 1) put forth by the Preferred Reporting Items for Systematic Reviews and Meta-Analyses (PRISMA) Statement [28].

### Information sources and literature search

Literature search strategies will be implemented by the research team (EVL, MM, IK, and MVZ) of the Research Centre of the University of Montreal Hospital Centre (CRCHUM), and an expert librarian (DZ) of the Documentation Center of the University of Montreal Hospital Centre (CHUM). Filters for bibliographic research will include publication date - from 1960 onwards - and languages, with consideration of English, French, Spanish, Portuguese and Chinese articles, conditional on the provision of an English abstract. We will use specific medical subject headings (MeSH) and text words to identify studies by searching MEDLINE (PubMed interface, 1960 onwards), EMBASE (OVID interface, 1974 - first year of indexation - onwards) and Cochrane Central (OVID interface, 1960 onwards). We will hand-search relevant abstracts in the Cochrane Neonatal Group, Cochrane Pregnancy and Childbirth Group, and Cochrane Public Health Group. As per the Peer Review of Electronic Search Strategies (PRESS) recommendation, we will include the ‘explode’ option to the Emtree terms in the EMBASE research [29]. The exact search strategy for MEDLINE, EMBASE, and Cochrane Central can be found in Additional file 1. We will search the gray literature, namely the following sources: Social Care Online; National Institute for Health and Clinical Excellence (NICE); System for information on Grey Literature in Europe (OpenSigle); National Guideline Clearing House; Health Development Agency; National Institutes of Health; Research Service Delivery and Organization Programme (SDO); Research Register for Social Care; Google Scholar and Open Grey. Furthermore, we will search official Websites of institutions active in the fields of maternal and child health and essential obstetric services, along with bibliographic references of retrieved articles and relevant reviews.

Our search strategy will combine terms related to the following categories: 1) postnatal or postpartum care; 2) utilization or accessibility; 3) determinants or inequalities; and 4) low- or middle-income countries. We will combine in a complete Endnote file all the scientific articles and reports retrieved through the identification phase, and then extract duplicates.

### Study inclusion criteria

#### Participants and setting

We will retrieve studies implemented in LMICs, as defined by The World Bank Group’s classification (see appendix of Additional file 2) [30], which study access to or utilization of PNC services by birthing women living in resource-strained settings.

#### Design

Our systematic review will include experimental studies covering randomized controlled trials (RCTs) and cluster-randomized trials (CRTs); quasi-experimental studies including quasi-randomized trials, controlled before-after studies (CBAs) and interrupted time series studies (ITSs); and observational studies including cohort, case–control and cross-sectional studies.

#### Outcomes

We will include studies reporting outcomes of postnatal/postpartum care services utilization. Although some studies underline that ‘postpartum’ refers to issues pertaining to the mother and ‘postnatal’ refers to those concerning the newborn or the baby, we will use the terms interchangeably, in accordance with WHO’s conceptualization (WHO, 2010) [9]. In a recent *WHO Technical Consultation on Postpartum and Postnatal Care (2010)*, a scientific panel agreed that adopting the single term ‘postnatal’ would aid clarity and should be used for all issues pertaining to the mother and the baby after birth [9]. Our primary outcome is the utilization of PNC services. Secondary outcomes include: 1) number of PNC visits; 2) timeliness of PNC services; 3) PNC location; and 4) nature, qualification and competence of the PNC attendant.

#### Determinants

Determinants of concern are: 1) socioeconomic status - assessed by income, expenditure, household characteristics and/or assets, occupational or contractual status [6,21,31,32] – and education (highest level of education completed, years of schooling, literacy) [33,34]; 2) geographic determinants (euclidian distance - km - to a health center, travel time, location - rural versus urban residence) [7,23]; and 3) demographic determinants: ethnicity, marital status, immigration status [32,33,35]. This list of determinants is retrieved from relevant scientific literature in essential obstetric services utilization in LMICs including original studies, systematic reviews and meta-analyses [6,7].

#### Results

We will consider quantitative results of the association between potential determinants and the utilization of PNC services. Published results must include an association measure, frequency ratio/difference, or statistical test comparing utilization of PNC services across two or more groups. If these results are not explicit, we have to be able to estimate them with the information provided in the paper. We will consider relative comparisons – for example, relative concentration index (RCI) or relative index of inequality (RII) - to a reference group, along with absolute differences in PNC services utilization, such as absolute concentration index (ACI) or slope index of inequality (SII). Such reported disparities will be useful in making comparisons over time or across geographical areas, populations or indicators, in light of the Centers for Disease Control and Prevention (CDC)’s guidelines [36,37]. Studies strictly reporting qualitative results on access to PNC are thus excluded. Within the same publication, results for the most recent year will be appraised if information exists for consecutive years. In the case of secondary analyses from national representative surveys such as the *Demographic and Health Surveys* (DHS) for consecutive years in the same country, we will only consider the most recent [7].

### Study selection procedure

#### Screening

A team of researchers, MM (Epidemiologist, PhD) and EVL (PhDc), will identify articles by first analyzing titles and abstracts for relevance and compliance with the selection criteria, based on research setting, study design, reported outcomes and determinants of interest. Relevant articles will be classified as: 1) included; 2) excluded; or 3) uncertain. After exclusion of records not relevant to the systematic review, full texts of selected abstracts (records categorized as included or uncertain) will be extracted systematically for further eligibility analysis.

#### Eligibility

Full-text screening will be conducted independently by the reviewers (MM and EVL) using a standardized form with explicit inclusion and exclusion criteria. Discrepancies will be resolved by discussion between the two reviewers, and persisting disagreement will be resolved by discussions with two experienced researchers (IK and MVZ). We will compute the inter-rater agreement using the intraclass correlation coefficient (ICC) [38].

### Data collection process

Reviewers will use an explicit data collection form to abstract data items, including but not limited to: study characteristics (country, setting, year of publication, study design, sample size); participants’ characteristics (mean age ± SD, parity, health literacy, women’s decision making power); outcomes (PNC utilization); and results of the association between PNC services and potential determinants. In cases where numerous publications report data originating from the same study, the latest outcomes of interest will be assessed. Missing data on key characteristics will be dealt with by contacting the study authors and through complementary research (for example, existence of user fees for maternal health services at the time of the study). Reviewers will systematically use a standardized data abstraction form [See Additional file 2]. To increase the reliability of data abstraction by the reviewers, a pilot test of the standardized form will be performed on a random sample, and the tool will be refined as necessary. MM and EVL will independently abstract the data, and discrepancies will be discussed with experienced reviewers (IK and MVZ).

### Scientific quality assessment

We will assess the scientific quality of selected studies to ensure internal validity of reported results and avoid analyzing spurious associations - confounded or biased – or type I statistical errors. We will use standardized quality assessment tools for specific types of designs to determine the methodological quality and the risk of bias of the included studies. To assess the quality of RCTs we will use the Cochrane Collaboration’s Risk of Bias Tool (CCRBT) [27]; for quasi-experimental designs, such as ITS and CBA studies, we will use the Cochrane Effective Practice and Organization of Practice (EPOC) Risk of Bias Tool [39]; and for cohort, case control and cross-sectional studies, we will use the Effective Public Health Practice Project (EPHPP) Quality Assessment Tool for Quantitative Studies, adapted to extend the criteria for selection bias assessment [40]. The latter instrument previously showed excellent inter-rater agreement for the final grade of studies [41], as well as adequate construct and content validity [42]. The EPHPP quality tool largely encompasses the principal quality items identified by the *Strengthening the Reporting of Observational Studies in Epidemiology (STROBE) Statement*[43]. Special attention will be provided to precise study objectives, explicit identification of the population studied, clear definitions of outcomes, independent factors, potential confounders and effect modifiers [44,45]. EVL and MM will independently appraise the scientific quality of the studies, and we will compute the inter-reviewer agreement using the ICC [38]. Discrepancies or uncertainties will be resolved through discussions with IK and MVZ. According to the methodological characteristics appraised, we will classify the studies’ scientific quality as either 1) Strong, 2) Moderate or 3) Weak.

### Search results

Evidence tables will be generated to descriptively summarize the included studies and results: 1) authors, 2) study design, 3) objectives, 4) setting, 5) population, 6) outcomes assessed, 7) determinants/predictors, 8) results and 9) scientific quality. Evidence tables will be stratified by countries’ income level (World Bank classification) to provide for different contextual characteristics of low- versus middle-income countries.

### Data synthesis

Where feasible, data will be combined to obtain a pooled measure of association evaluating PNC services inequalities, through meta-analyses conducted by using The Cochrane Group’s Review Manager Software (RevMan 5.1) [46]. Data will be analyzed along subsets defined by the countries' income level and grouped by determinants of PNC services utilization (socioeconomic, geographic, demographic). Due consideration will be given to heterogeneity (I^2^ statistic) and corresponding analysis (fixed versus random-effects models; meta-regression, if necessary). Data synthesis will be stratified and presented separately for experimental, quasi-experimental and observational studies. Depending on the number of studies, we will further stratify observational studies according to design (cohort, case–control, cross-sectional) and/or association measure - odds ratio, risk ratio, incidence rate ratio, hazard ratio, and prevalence ratio - exploring potential heterogeneity. Where feasible, we will carry out separate meta-analyses of adjusted versus non-adjusted (or insufficiently adjusted) association measures. Should we notice conditions that impede meta-analysis, we will synthetize the data narratively to provide for PNC services inequalities. Particular attention will be paid to assessing results in light of study settings to ensure proper contextualization of evidence and relevance for policy planning purposes in LMICs. Results will be reported according to the PRISMA Statement, with a focus on health equity (PRISMA-Equity 2012 Extension) [47].

### Consent

Oral consent was obtained from the women for the publication of the accompanying image.

## Discussion

This systematic review will provide: 1) knowledge on existing inequalities and unmet needs for PNC services in LMICs; 2) pragmatic recommendations to health policy planners for improving access to, and utilization of, quality PNC in LMICs; and 3) an overview of knowledge gaps and future research needs. Results of the systematic review will be published in a peer-reviewed international journal and presented at conferences and symposia in relevant fields (for example, global health, health policy and planning, health systems, healthcare equity). Further knowledge dissemination will involve communicating results to the governments of LMICs and to organizations active in promoting access to maternal and child health services (for example, WHO, Family Care International). The utmost relevance of systematic reviews to inform health systems policymaking is increasingly recognized [48]. Tugwell *et al.* (2010) underlined that a focus on health equity in systematic reviews improves their relevance for public policy making [37]. Welch *et al.* (2012) stressed that systematic reviews are a valuable source of scientific evidence on inequities in health outcomes, resource allocation and use [47]. Our review will hence supply evidence to health policy planners with the objective of decreasing inequalities in maternal and child health indicators and promoting universal coverage of essential obstetric care services. Knowledge thus created may help promote equitable access to postnatal services as a fundamental element of the continuum of care essential to reduce maternal and neonatal mortality and morbidity. This protocol was registered with the Prospero database (registration number: CRD42013004661).

## Abbreviations

ANC: Antenatal care; CBAs: Controlled before-after studies; CDC: Centers for disease control and prevention; DHS: Demographic and health surveys; EBF: Exclusive breastfeeding; EPHPP: Effective public health practice project; ICC: Intraclass correlation coefficient; ITS: Interrupted time series; LBW: Low birth weight; LMICs: Low- and middle-income countries; MeSH: Medical subject headings; PNC: Postnatal care; PRISMA: Preferred reporting items for systematic reviews and meta-analyses; RCT: Randomized controlled trial; SBA: Skilled birth attendant; WHO: World Health Organization.

## Competing interests

The authors declare that they have no competing interests.

## Authors’ contributions

EVL, IK, MM and MVZ contributed to the conception and design of the review. EVL and DZ developed the search strategies. EVL drafted the manuscript. IK, MM and MVZ were actively involved in critically revising the protocol for important intellectual content. DZ made a substantial contribution to the ‘Information sources and literature search’ section, and to Additional file 1. All authors read and approved the final manuscript.

## Supplementary Material

Additional file 1 Search strategy MEDLINE, EMBASE, and Cochrane Central. Click here for file

Additional file 2 Data collection form. Click here for file
