# Supplementary material for: Protocol for a systematic review on inequalities in postnatal care services utilization in low- and middle-income countries
Source: Syst Rev. 2013 Jul 6;2:55. doi: 10.1186/2046-4053-2-55 (PMC3717005; doi:10.1186/2046-4053-2-55)
Supplement: Additional file 1 — Search strategy MEDLINE, EMBASE, and Cochrane Central. [file 2046-4053-2-55-S1.docx]

**Search Strategy - Protocol for a Systematic Review on Inequalities in Postnatal Care Services Utilization in Low- and Middle-Income Countries**

**1. MEDLINE (PubMed interface, 1960 onwards)**

(("postnatal care"[MeSH Terms] OR ("postnatal"[All Fields] AND "care"[All Fields]) OR "postnatal care"[All Fields]) OR ("postnatal care"[MeSH Terms] OR ("postnatal"[All Fields] AND "care"[All Fields]) OR "postnatal care"[All Fields] OR ("postpartum"[All Fields] AND "care"[All Fields]) OR "postpartum care"[All Fields]) OR ("perinatal care"[MeSH Terms] OR ("perinatal"[All Fields] AND "care"[All Fields]) OR "perinatal care"[All Fields]) OR (("postpartum period"[MeSH Terms] OR ("postpartum"[All Fields] AND "period"[All Fields]) OR "postpartum period"[All Fields] OR "puerperium"[All Fields]) AND care[All Fields]) OR (postnatal[All Fields] AND follow[All Fields] AND up[All Fields]) OR (("postpartum period"[MeSH Terms] OR ("postpartum"[All Fields] AND "period"[All Fields]) OR "postpartum period"[All Fields] OR "postpartum"[All Fields]) AND follow[All Fields] AND up[All Fields]) OR (("postpartum period"[MeSH Terms] OR ("postpartum"[All Fields] AND "period"[All Fields]) OR "postpartum period"[All Fields] OR "puerperium"[All Fields]) AND follow[All Fields] AND up[All Fields]) OR (postnatal[All Fields] AND monitoring[All Fields]) OR (("postpartum period"[MeSH Terms] OR ("postpartum"[All Fields] AND "period"[All Fields]) OR "postpartum period"[All Fields] OR "postpartum"[All Fields]) AND monitoring[All Fields]) OR (("postpartum period"[MeSH Terms] OR ("postpartum"[All Fields] AND "period"[All Fields]) OR "postpartum period"[All Fields] OR "puerperium"[All Fields]) AND monitoring[All Fields]) OR ("prenatal care"[MeSH Terms] OR ("prenatal"[All Fields] AND "care"[All Fields]) OR "prenatal care"[All Fields])))

**AND**

(access[All Fields] OR accessibility[All Fields] OR ("utilization"[Subheading] OR "utilization"[All Fields]) OR utilisation[All Fields] OR ("utilization"[Subheading] OR "utilization"[All Fields] OR "use"[All Fields]) OR "coverage"[All Fields] OR (("health services"[MeSH Terms] OR ("health"[All Fields] AND "services"[All Fields]) OR "health services"[All Fields] OR ("health"[All Fields] AND "service"[All Fields]) OR "health service"[All Fields]) AND access[All Fields]) OR (("health services"[MeSH Terms] OR ("health"[All Fields] AND "services"[All Fields]) OR "health services"[All Fields] OR ("health"[All Fields] AND "service"[All Fields]) OR "health service"[All Fields]) AND ("utilization"[Subheading] OR "utilization"[All Fields])) OR (("health services"[MeSH Terms] OR ("health"[All Fields] AND "services"[All Fields]) OR "health services"[All Fields] OR ("health"[All Fields] AND "service"[All Fields]) OR "health service"[All Fields]) AND accessibility[All Fields]) OR ("health services accessibility"[MeSH Terms] OR ("health"[All Fields] AND "services"[All Fields] AND "accessibility"[All Fields]) OR "health services accessibility"[All Fields] OR ("access"[All Fields] AND "health"[All Fields] AND "care"[All Fields]) OR "access to health care"[All Fields]) OR (("delivery of health care"[MeSH Terms] OR ("delivery"[All Fields] AND "health"[All Fields] AND "care"[All Fields]) OR "delivery of health care"[All Fields] OR ("health"[All Fields] AND "care"[All Fields]) OR "health care"[All Fields]) AND ("utilization"[Subheading] OR "utilization"[All Fields])) OR (("delivery of health care"[MeSH Terms] OR ("delivery"[All Fields] AND "health"[All Fields] AND "care"[All Fields]) OR "delivery of health care"[All Fields] OR ("health"[All Fields] AND "care"[All Fields]) OR "health care"[All Fields]) AND accessibility[All Fields]) OR ("health services accessibility"[MeSH Terms] OR ("health"[All Fields] AND "services"[All Fields] AND "accessibility"[All Fields]) OR "health services accessibility"[All Fields] OR ("accessibility"[All Fields] AND "health"[All Fields] AND "services"[All Fields]) OR "accessibility of health services"[All Fields]) OR (accessibility[All Fields] AND ("health services"[MeSH Terms] OR ("health"[All Fields] AND "services"[All Fields]) OR "health services"[All Fields])) OR (access[All Fields] AND ("health services"[MeSH Terms] OR ("health"[All Fields] AND "services"[All Fields]) OR "health services"[All Fields])) OR (access[All Fields] AND ("health services"[MeSH Terms] OR ("health"[All Fields] AND "services"[All Fields]) OR "health services"[All Fields])) OR (accessibility[All Fields] AND ("delivery of health care"[MeSH Terms] OR ("delivery"[All Fields] AND "health"[All Fields] AND "care"[All Fields]) OR "delivery of health care"[All Fields] OR ("health"[All Fields] AND "care"[All Fields]) OR "health care"[All Fields])) OR (("health facilities"[MeSH Terms] OR ("health"[All Fields] AND "facilities"[All Fields]) OR "health facilities"[All Fields] OR ("health"[All Fields] AND "facility"[All Fields]) OR "health facility"[All Fields]) AND ("delivery, obstetric"[MeSH Terms] OR ("delivery"[All Fields] AND "obstetric"[All Fields]) OR "obstetric delivery"[All Fields] OR "delivery"[All Fields])))

**AND**

(("socioeconomic factors"[MeSH Terms] OR ("socioeconomic"[All Fields] AND "factors"[All Fields]) OR "socioeconomic factors"[All Fields] OR "inequality"[All Fields]) OR ("socioeconomic factors"[MeSH Terms] OR ("socioeconomic"[All Fields] AND "factors"[All Fields]) OR "socioeconomic factors"[All Fields] OR "inequalities"[All Fields]) OR equity[All Fields] OR inequity[All Fields] OR inequities[All Fields] OR socio-economic[All Fields] OR ("socioeconomic factors"[MeSH Terms] OR ("socioeconomic"[All Fields] AND "factors"[All Fields]) OR "socioeconomic factors"[All Fields] OR "inequalities"[All Fields]) OR (socio-economic[All Fields] AND ("socioeconomic factors"[MeSH Terms] OR ("socioeconomic"[All Fields] AND "factors"[All Fields]) OR "socioeconomic factors"[All Fields] OR "inequality"[All Fields])) OR (socio-economic[All Fields] AND inequities[All Fields]) OR (socio-economic[All Fields] AND inequity[All Fields]) OR (socioeconomic[All Fields] AND ("socioeconomic factors"[MeSH Terms] OR ("socioeconomic"[All Fields] AND "factors"[All Fields]) OR "socioeconomic factors"[All Fields] OR "inequalities"[All Fields])) OR (socioeconomic[All Fields] AND ("socioeconomic factors"[MeSH Terms] OR ("socioeconomic"[All Fields] AND "factors"[All Fields]) OR "socioeconomic factors"[All Fields] OR "inequality"[All Fields])) OR (socioeconomic[All Fields] AND inequities[All Fields]) OR (socioeconomic[All Fields] AND inequity[All Fields]) OR (unmet[All Fields] AND need[All Fields]) OR barrier[All Fields] OR ("income"[MeSH Terms] OR "income"[All Fields]) OR ("socioeconomic factors"[MeSH Terms] OR ("socioeconomic"[All Fields] AND "factors"[All Fields]) OR "socioeconomic factors"[All Fields] OR "socioeconomics"[All Fields]) OR geographic[All Fields] OR exclusion[All Fields] OR ("poverty"[MeSH Terms] OR "poverty"[All Fields]) OR vulnerability[All Fields] OR marginalized[All Fields] OR vulnerable[All Fields] OR marginalization[All Fields] OR ("social distance"[MeSH Terms] OR ("social"[All Fields] AND "distance"[All Fields]) OR "social distance"[All Fields] OR ("social"[All Fields] AND "exclusion"[All Fields]) OR "social exclusion"[All Fields]) OR (social[All Fields] AND stratification[All Fields]) OR gradient[All Fields] OR determinant[All Fields] OR predictor[All Fields] OR propension[All Fields] OR ("socioeconomic factors"[MeSH Terms] OR ("socioeconomic"[All Fields] AND "factors"[All Fields]) OR "socioeconomic factors"[All Fields]) OR ("risk factors"[MeSH Terms] OR ("risk"[All Fields] AND "factors"[All Fields]) OR "risk factors"[All Fields] OR ("risk"[All Fields] AND "factor"[All Fields]) OR "risk factor"[All Fields]) OR residence[All Fields] OR location[All Fields] OR ("ethnology"[Subheading] OR "ethnology"[All Fields] OR "ethnicity"[All Fields] OR "ethnology"[MeSH Terms] OR "ethnicity"[All Fields] OR "ethnic groups"[MeSH Terms] OR ("ethnic"[All Fields] AND "groups"[All Fields]) OR "ethnic groups"[All Fields]) OR (("emigration and immigration"[MeSH Terms] OR ("emigration"[All Fields] AND "immigration"[All Fields]) OR "emigration and immigration"[All Fields] OR "immigration"[All Fields]) AND status[All Fields])))

**AND**

(((("developing countries"[MeSH Terms] OR ("developing"[All Fields] AND "countries"[All Fields]) OR "developing countries"[All Fields]) OR ("developing countries"[MeSH Terms] OR ("developing"[All Fields] AND "countries"[All Fields]) OR "developing countries"[All Fields] OR ("developing"[All Fields] AND "country"[All Fields]) OR "developing country"[All Fields]) OR ("developing countries"[MeSH Terms] OR ("developing"[All Fields] AND "countries"[All Fields]) OR "developing countries"[All Fields] OR ("underdeveloped"[All Fields] AND "countries"[All Fields]) OR "underdeveloped countries"[All Fields]) OR ("developing countries"[MeSH Terms] OR ("developing"[All Fields] AND "countries"[All Fields]) OR "developing countries"[All Fields] OR ("underdeveloped"[All Fields] AND "country"[All Fields]) OR "underdeveloped country"[All Fields]) OR (emergent[All Fields] AND countries[All Fields]) OR (emergent[All Fields] AND country[All Fields]) OR ("developing countries"[MeSH Terms] OR ("developing"[All Fields] AND "countries"[All Fields]) OR "developing countries"[All Fields] OR ("developing"[All Fields] AND "nation"[All Fields]) OR "developing nation"[All Fields]) OR (underdeveloped[All Fields] AND "nation"[All Fields])) OR (emergent[All Fields] AND "nation"[All Fields]) OR ("africa"[MeSH Terms] OR "africa"[All Fields]) OR (("poverty"[MeSH Terms] OR "poverty"[All Fields] OR ("low"[All Fields] AND "income"[All Fields]) OR "low income"[All Fields]) AND countries[All Fields]) OR (("poverty"[MeSH Terms] OR "poverty"[All Fields] OR ("low"[All Fields] AND "income"[All Fields]) OR "low income"[All Fields]) AND country[All Fields]) OR (middle[All Fields] AND ("income"[MeSH Terms] OR "income"[All Fields]) AND countries[All Fields]) OR (middle[All Fields] AND ("income"[MeSH Terms] OR "income"[All Fields]) AND country[All Fields]) OR (("poverty"[MeSH Terms] OR "poverty"[All Fields] OR "poor"[All Fields]) AND setting[All Fields]) OR (("health resources"[MeSH Terms] OR ("health"[All Fields] AND "resources"[All Fields]) OR "health resources"[All Fields] OR "resource"[All Fields]) AND limited[All Fields] AND setting[All Fields]) OR (("health resources"[MeSH Terms] OR ("health"[All Fields] AND "resources"[All Fields]) OR "health resources"[All Fields] OR "resource"[All Fields]) AND scarce[All Fields] AND setting[All Fields]) OR (resource-limited[All Fields] AND setting[All Fields]) OR (resource-scarce[All Fields] AND setting[All Fields]) OR (("poverty"[MeSH Terms] OR "poverty"[All Fields] OR "poor"[All Fields]) AND country[All Fields]) OR (("poverty"[MeSH Terms] OR "poverty"[All Fields] OR ("low"[All Fields] AND "income"[All Fields]) OR "low income"[All Fields]) AND "nation"[All Fields]) OR (middle[All Fields] AND ("income"[MeSH Terms] OR "income"[All Fields]) AND "nation"[All Fields]) OR (third[All Fields] AND "world"[All Fields]) OR ("middle east"[MeSH Terms] OR ("middle"[All Fields] AND "east"[All Fields]) OR "middle east"[All Fields]) OR ("india"[MeSH Terms] OR "india"[All Fields]) OR ("asia"[MeSH Terms] OR "asia"[All Fields]) OR ("europe, eastern"[MeSH Terms] OR ("europe"[All Fields] AND "eastern"[All Fields]) OR "eastern europe"[All Fields] OR ("eastern"[All Fields] AND "europe"[All Fields])) OR ("philippines"[MeSH Terms] OR "philippines"[All Fields]) OR ("indonesia"[MeSH Terms] OR "indonesia"[All Fields]) OR ("latin america"[MeSH Terms] OR ("latin"[All Fields] AND "america"[All Fields]) OR "latin america"[All Fields]) OR ("south america"[MeSH Terms] OR ("south"[All Fields] AND "america"[All Fields]) OR "south america"[All Fields]) OR ("central america"[MeSH Terms] OR ("central"[All Fields] AND "america"[All Fields]) OR "central america"[All Fields]) OR ("china"[MeSH Terms] OR "china"[All Fields]) OR ("russia"[MeSH Terms] OR "russia"[All Fields]))

**AND**

("1960/01/01"[PDAT]: "2013/12/31"[PDAT])

**2. EMBASE (OVID interface)**

As per the Peer Review of Electronic Search Strategies (PRESS) recommendation, we added the “explode” option to the Emtree terms - MeSH equivalent - among the EMBASE research. To explode a subject heading involves including a selected subject heading and all of the narrower terms that are below it in the hierarchy (subject headings are arranged hierarchically in many thesauri).

Database: Embase <1974 to 2013 June 06>

Search Strategy:

--------------------------------------------------------------------------------

1 access.af. (239045)

2 accessibility.af. (27386)

3 utili#ation$.af. (260218)

4 health care access/ (33742)

5 coverage$.af. (72436)

6 health service$ access.af. (168)

7 health service$ utilization$.af. (2125)

8 health care utilization/ (36105)

9 health service$ accessibility.af. (571)

10 access to health care.af. (4360)

11 health care utilization$.af. (37525)

12 health care delivery/ (122662)

13 health care accessibility.af. (65)

14 accessibility of health service$.af. (122)

15 accessibility to health service$.af. (106)

16 access to health service$.af. (1042)

17 access of health service$.af. (1024)

18 accessibility to health care.af. (165)

19 health care facility/ (49668)

20 health facility delivery.af. (18)

21 or/1-20 (704018)

22 exp socioeconomics/ or exp social status/ or exp social class/ or exp health disparity/ or exp health status/ (322942)

23 inequality.af. (10141)

24 inequalities.af. (10169)

25 equity.af. (10231)

26 inequity.af. (1652)

27 inequities.af. (2517)

28 socio-economic$.af. (23871)

29 socio-economic inequality.af. (53)

30 socio-economic inequalities.af. (245)

31 socioeconomic inequality.af. (201)

32 socioeconomic inequalities.af. (717)

33 unmet need$.af. (5874)

34 barrier$.af. (195993)

35 income.af. (81172)

36 socioeconomics.af. (106420)

37 geographic.af. (185684)

38 exclusion.af. (80576)

39 poverty.af. (34969)

40 exp poverty/ (28104)

41 vulnerability.af. (37242)

42 marginalized.af. (1738)

43 vulnerable.af. (51515)

44 exp vulnerable population/ (5546)

45 marginalization.af. (851)

46 social exclusion.af. (974)

47 exp social exclusion/ (72)

48 gradient.af. (162338)

49 determinant.af. (75598)

50 predictor.af. (141269)

51 propension.af. (54)

52 socioeconomics factor$.af. (13)

53 exp risk factor/ (575916)

54 risk factor$.af. (745344)

55 residence.af. (37042)

56 location.af. (254146)

57 exp ethnic group/ or exp ethnicity/ (208878)

58 ethnicity.af. (55128)

59 exp immigration/ (3859)

60 immigration status.af. (345)

61 or/22-60 (2308415)

62 exp postnatal care/ (71705)

63 postnatal care.af. (4856)

64 exp puerperium/ (39796)

65 postpartum care.af. (484)

66 exp perinatal care/ (34089)

67 perinatal care.af. (8652)

68 puerperium care.af. (5)

69 postnatal follow up.af. (340)

70 postnatal followup.af. (7)

71 postpartum follow up.af. (155)

72 postpartum followup.af. (5)

73 puerperium follow up.af. (3)

74 puerperium followup.af. (0)

75 postnatal monitoring.af. (14)

76 postpartum monitoring.af. (16)

77 puerperium monitoring.af. (1)

78 exp prenatal care/ (106298)

79 prenatal care.af. (28134)

80 or/62-79 (202473)

81 exp developing country/ (74556)

82 developing country.af. (76868)

83 developing countries.af. (41657)

84 underdeveloped country.af. (63)

85 underdeveloped countries.af. (832)

86 emergent countries.af. (18)

87 emergent country.af. (3)

88 developing nation$.af. (2122)

89 underdeveloped nation$.af. (66)

90 emergent nation$.af. (5)

91 exp Africa/ (201797)

92 africa.af. (177755)

93 low income country.af. (329)

94 low income countries.af. (2505)

95 middle income countries.af. (3082)

96 middle income country.af. (319)

97 poor setting$.af. (1734)

98 resource$ limited setting.af. (467)

99 resource$ scarce setting.af. (4)

100 poor country.af. (147)

101 poor countries.af. (1702)

102 low income nation$.af. (43)

103 middle income nation$.af. (36)

104 third world.af. (3230)

105 exp Middle East/ (101013)

106 middle east.af. (12394)

107 india.af. (513843)

108 exp India/ (82864)

109 asia.af. (87500)

110 exp Asia/ (570433)

111 eastern europe.af. (7407)

112 exp Philippines/ (6711)

113 exp Eastern Europe/ (165099)

114 Philippines.af. (14764)

115 exp Indonesia/ (8054)

116 indonesia.af. (16298)

117 exp "South and Central America"/ (131019)

118 Latin America.af. (9750)

119 South America.af. (15831)

120 Central America.af. (18210)

121 China.af. (982934)

122 Russia.af. (173702)

123 exp China/ (85318)

124 exp Russian Federation/ (44905)

125 exp Central America/ (15945)

126 exp South America/ (111821)

127 or/81-126 (2639353)

128 21 and 61 and 80 and 127 (2172)

129 limit 128 to embase (1445)

***************************

**3. Cochrane Central (OVID interface)**

Databases: OVID Evidence-Based Medicine Reviews (EBMR). Cochrane Database of Systematic Reviews <2005 to April 2013>, EBM Reviews - ACP Journal Club <1991 to May 2013>, EBM Reviews - Database of Abstracts of Reviews of Effects <1991 to May 2013>, EBM Reviews - Cochrane Central Register of Controlled Trials <1991 to May 2013>, EBM Reviews - Cochrane Methodology Register <1960 to present>, EBM Reviews - Health Technology Assessment <2001 to present>, EBM Reviews - NHS Economic Evaluation Database <1995 to present>

We specifically hand-searched relevant abstracts in the following review groups : Cochrane Neonatal Group (377); Cochrane Pregnancy and Childbirth Group (596); and Cochrane Public Health Group (29).

Search Strategy:

--------------------------------------------------------------------------------

1 access.af. (7661)

2 accessibility.af. (1065)

3 utili#ation$.af. (7229)

4 health care access.af. (70)

5 coverage$.af. (2469)

6 health service$ access.af. (9)

7 health service$ utilization$.af. (222)

8 health care utilization/ (1)

9 health service$ accessibility.af. (495)

10 access to health care.af. (125)

11 health care utilization$.af. (484)

12 health care delivery/ (470)

13 health care accessibility.af. (2)

14 accessibility of health service$.af. (3)

15 accessibility to health service$.af. (3)

16 access to health service$.af. (40)

17 access of health service$.af. (40)

18 accessibility to health care.af. (7)

19 health care facility.af. (77)

20 (health adj6 facility adj6 delivery).af. (14)

21 or/1-20 (17172)

22 exp socioeconomics/ or exp social status/ or exp social class/ or exp health disparity/ or exp health status/ (4844)

23 inequality.af. (163)

24 inequalities.af. (248)

25 equity.af. (284)

26 inequity.af. (48)

27 inequities.af. (49)

28 socio-economic$.af. (737)

29 (socio-economic adj6 inequality).af. (2)

30 (socio-economic adj6 inequalities).af. (6)

31 (socioeconomic adj6 inequality).af. (1)

32 socioeconomic inequalities.af. (14)

33 unmet need$.af. (199)

34 barrier$.af. (5236)

35 income.af. (3425)

36 socioeconomics.af. (65)

37 geographic.af. (1112)

38 exclusion.af. (13533)

39 poverty.af. (1231)

40 exp poverty/ (854)

41 vulnerability.af. (694)

42 marginalized.af. (46)

43 vulnerable.af. (1506)

44 exp vulnerable population/ (74)

45 marginalization.af. (10)

46 social exclusion.af. (49)

47 social exclusion.af. (49)

48 gradient.af. (1760)

49 determinant.af. (1699)

50 predictor.af. (5631)

51 propension.af. (1)

52 (socioeconomics adj3 factor$).af. (3)

53 exp risk factor/ (15569)

54 risk factor$.af. (29333)

55 residence.af. (1350)

56 location.af. (7567)

57 exp ethnic group/ or exp ethnicity/ (2375)

58 ethnicity.af. (2090)

59 exp immigration/ (68)

60 immigration status.af. (6)

61 or/22-60 (68355)

62 exp postnatal care/ (243)

63 postnatal care.af. (355)

64 exp puerperium/ (898)

65 postpartum care.af. (91)

66 exp perinatal care/ (288)

67 perinatal care.af. (228)

68 (puerperium adj3 care).af. (9)

69 postnatal follow up.af. (9)

70 postpartum follow up.af. (26)

71 puerperium follow up.af. (1)

72 (postnatal adj6 monitoring).af. (4)

73 (postpartum adj6 monitoring).af. (10)

74 (puerperium adj3 monitoring).af. (1)

75 exp prenatal care/ (851)

76 prenatal care.af. (1231)

77 or/62-76 (2558)

78 exp developing country/ (455)

79 developing country.af. (359)

80 developing countries.af. (2523)

81 (underdeveloped adj3 country).af. (2)

82 underdeveloped countries.af. (23)

83 (emergent adj6 countries).af. (0)

84 (emergent adj4 country).af. (0)

85 developing nation$.af. (85)

86 underdeveloped nation$.af. (2)

87 (emergent adj3 nation$).af. (1)

88 exp Africa/ (3706)

89 africa.af. (4126)

90 low income country.af. (50)

91 low income countries.af. (381)

92 middle income countries.af. (491)

93 middle income country.af. (59)

94 poor setting$.af. (172)

95 resource$ limited setting.af. (30)

96 (resource$ adj3 scarce adj3 setting).af. (0)

97 poor country.af. (4)

98 poor countries.af. (97)

99 low income nation$.af. (5)

100 middle income nation$.af. (6)

101 third world.af. (89)

102 exp Middle East/ (1459)

103 middle east.af. (219)

104 india.af. (6357)

105 exp India/ (1090)

106 asia.af. (1649)

107 exp Asia/ (8820)

108 eastern europe.af. (101)

109 exp Philippines/ (103)

110 exp Eastern Europe/ (889)

111 Philippines.af. (395)

112 exp Indonesia/ (202)

113 indonesia.af. (535)

114 South.mp. and Central America.af. [mp=ti, ab, tx, kw, ct, ot, sh, hw] (51)

115 Latin America.af. (411)

116 South America.af. (406)

117 Central America.af. (91)

118 China.af. (11504)

119 Russia.af. (760)

120 exp China/ (1619)

121 Russian Federation.af. (81)

122 exp Central America/ (175)

123 exp South America/ (1228)

124 or/78-123 (35012)

125 and/21,61,77,124 (94)

126 remove duplicates from 125 (94)

***************************
