# Supplementary material for: Protocol for a systematic review on inequalities in postnatal care services utilization in low- and middle-income countries
Source: Syst Rev. 2013 Jul 6;2:55. doi: 10.1186/2046-4053-2-55 (PMC3717005; doi:10.1186/2046-4053-2-55)
Supplement: Additional file 2 — Data collection form. [file 2046-4053-2-55-S2.doc]

# Data collection form

## Notes:

- Be consistent in the order and style you use to describe the information for each report.
- Record any missing information as unclear or not described, to make it clear that the information was not found in the study report(s), not that you forgot to extract it.

| Review title | Systematic Review on Inequalities in Postnatal Care Services Utilization in Low- and Middle-Income Countries |
| --- | --- |
| Study ID *(surname of first author and year first full report of study was published e.g. Smith 2001)* |  |
| Notes | |

# General Information

| Date form completed *(dd/mm/yyyy)* |  |
| --- | --- |
| Name/ID of person extracting data |  |
| Reference citation (e.g. Medline) |  |
| Study author contact details |  |
| Publication type  *(e.g. full report, abstract, letter)* |  |
| Notes: | |

# Study eligibility

| Study Characteristics | Eligibility criteria | | Eligibility criteria met? | | | Location in text or source *(pg & ¶/fig/table)* |
| --- | --- | --- | --- | --- | --- | --- |
|  |  |  | Yes | No | Unclear |  |
| Type of study | Experimental study including randomized controlled trials (RCTs) or cluster-randomized trials (CRTs). | |  |  |  |  |
|  | Quasi-experimental studies including quasi-randomized trials, controlled before-after studies (CBAs) and interrupted time series studies (ITSs). | |  |  |  |  |
|  | Observational studies including cohort, case-control and cross-sectional studies. | |  |  |  |  |
| Participants and setting | Birthing women from low- and middle-income countries, as defined by The World Bank Group’s classification (*see Appendix for countries’ income groups*) | |  |  |  |  |
| Types of intervention | Postnatal care services | |  |  |  |  |
| Types of comparison | PNC services users vs non-users | |  |  |  |  |
| Types of outcome measures | Primary outcome:  Postnatal care services utilization  Secondary outcomes:  1) Number of PNC visits;  2) Timeliness of PNC services;  3) PNC location, and  4) Nature, qualification and competence of PNC attendant. | |  |  |  |  |
| Types of determinants | Determinants of concern are:  1) Socioeconomic status - assessed by income, expenditure, household characteristics and/or assets, occupational or contractual status – and education (highest level of education completed, years of schooling, literacy);  2) Geographic (euclidian distance - km - to a health center, travel time, location - rural vs. urban residence);  3) Demographic (ethnicity, marital status, immigration status). | |  |  |  |  |
| Results | Quantitative results of the association between potential determinants and postnatal care services utilization | |  |  |  |  |
| INCLUDE | | EXCLUDE | | | | |
| Reason for exclusion |  | | | | | |
| Notes: | | | | | | |

**DO NOT PROCEED IF STUDY EXCLUDED FROM REVIEW**

# Characteristics of included studies

## Methods

|  | **Descriptions as stated in report/paper** | | **Location in text or source** *(pg & ¶/fig/table)* |
| --- | --- | --- | --- |
| **Aim of study** |  | |  |
| **Design** |  | |  |
| **Unit of observation** |  | |  |
| **Start date** |  | |  |
| **End date** |  | |  |
| **Duration of participation**  *(from recruitment to last follow-up)* |  | |  |
| **Ethical approval needed/ obtained for study** | Yes No Unclear |  |  |
| **Notes:** | | | |

## Participants and PNC Utilization

|  | Description | | Location in text or source *(pg & ¶/fig/table)* |
| --- | --- | --- | --- |
| Population description  *(from which study participants are drawn)* |  | |  |
| Setting and context  *(including but not limited to healthcare system characteristics and health financing - e.g. user fees or financial coverage of PNC services - as well as social context, location).* |  | |  |
| Inclusion criteria |  | |  |
| Exclusion criteria |  | |  |
| Method of recruitment of participants *(e.g. phone, mail, clinic patients)* |  | |  |
| Informed consent obtained | Yes No Unclear |  |  |
| Total no. of subjects |  | |  |
| Clusters  *(if applicable, no., type, no. people per cluster)* |  | |  |
| Baseline imbalances  *(if applicable)* |  | |  |
| Withdrawals and exclusions |  | |  |
| Number of total person-years (if applicable) |  | |  |
| Missing data |  | |  |
| Outcome(s)  Definition, measure & classification | Primary outcome - Postnatal care services utilization | |  |
|  | Secondary outcomes  1) Number of PNC visits  2) Timeliness of PNC services  3) PNC location  4) Nature, qualification and competence of PNC attendant | |  |
| Determinants | Socioeconomic | |  |
|  | Geographic | |  |
|  | Demographic | |  |
| Confounding factors/ effect modifiers accounted for |  | |  |
| Results  (specify, e.g. OR, RR, IRR)  (specify the reference group) | Crude | |  |
|  | Adjusted | |  |
| Authors’ reported limitations of study’s methods/results |  | |  |
| Scientific quality (specify tool, e.g. modified EPHPP tool) |  | |  |
| Notes: | | | |

## Other information

| **Study funding sources**  *(including role of funders)* |  |  |
| --- | --- | --- |
| **Possible conflicts of interest**  *(for study authors)* |  |  |
|  | **Description as stated in report/paper** | **Location in text or source** |
| **Key conclusions of study authors** |  |  |
| **References to other relevant studies** |  |  |
| **Correspondence required for further study information** *(from whom, what and when)* |  | |
| **Notes:** | | |

**Appendix**

*The World Bank Group’s classification of countries by income groups*

Available from:

<http://data.worldbank.org/about/country-classifications/country-and-lending-groups>

**Low-income economies**

| Afghanistan | Gambia, The | Mozambique |
| --- | --- | --- |
| Bangladesh | Guinea | Myanmar |
| Benin | Guinea-Bisau | Nepal |
| Burkina Faso | Haiti | Niger |
| Burundi | Kenya | Rwanda |
| Cambodia | Korea, Dem Rep. | Sierra Leone |
| Central African Republic | Kyrgyz Republic | Somalia |
| Chad | Liberia | Tajikistan |
| Comoros | Madagascar | Tanzania |
| Congo, Dem. Rep | Malawi | Togo |
| Eritrea | Mali | Uganda |
| Ethiopia | Mauritania | Zimbabwe |

**Lower-middle-income economies**

| Albania | Indonesia | Samoa |
| --- | --- | --- |
| Armenia | India | São Tomé and Principe |
| Belize | Iraq | Senegal |
| Bhutan | Kiribati | Solomon Islands |
| Bolivia | Kosovo | South Sudan |
| Cameroon | Lao PDR | Sri Lanka |
| Cape Verde | Lesotho | Sudan |
| Congo, Rep. | Marshall Islands | Swaziland |
| Côte d'Ivoire | Micronesia, Fed. Sts. | Syrian Arab Republic |
| Djibouti | Moldova | Timor-Leste |
| Egypt, Arab Rep. | Mongolia | Tonga |
| El Salvador | Morocco | Ukraine |
| Fiji | Nicaragua | Uzbekistan |
| Georgia | Nigeria | Vanuatu |
| Ghana | Pakistan | Vietnam |
| Guatemala | Papua New Guinea | West Bank and Gaza |
| Guyana | Paraguay | Yemen, Rep. |
| Honduras | Philippines | Zambia |

**Upper-middle-income economies**

| Angola | Ecuador | Palau |
| --- | --- | --- |
| Algeria | Gabon | Panama |
| American Samoa | Grenada | Peru |
| Antigua and Barbuda | Iran, Islamic Rep. | Romania |
| Argentina | Jamaica | Russian Federation |
| Azerbaijan | Jordan | Serbia |
| Belarus | Kazakhstan | Seychelles |
| Bosnia and Herzegovina | Latvia | South Africa |
| Botswana | Lebanon | St. Lucia |
| Brazil | Libya | St. Vincent and the Grenadines |
| Bulgaria | Lithuania | Suriname |
| Chile | Macedonia, FYR | Thailand |
| China | Malaysia | Tunisia |
| Colombia | Maldives | Turkey |
| Costa Rica | Mauritius | Turkmenistan |
| Cuba | Mexico | Tuvalu |
| Dominica | Montenegro | Uruguay |
| Dominican Republic | Namibia | Venezuela, RB |
